# The Emissions Fractions Approach to Assessing the Long-Range Transport Potential of Organic Chemicals

**DOI:** 10.1021/acs.est.2c03047

**Published:** 2022-08-11

**Authors:** Knut Breivik, Michael S. McLachlan, Frank Wania

**Affiliations:** † Norwegian Institute for Air Research, P.O. Box 100, NO-2027 Kjeller, Norway; ‡ Department of Chemistry, University of Oslo, P.O. Box 1033, NO-0315 Oslo, Norway; § Department of Environmental Science, Stockholm University, SE-106 91 Stockholm, Sweden; ∥ Department of Physical and Environmental Sciences, University of Toronto Scarborough, 1265 Military Trail, Toronto, Ontario M1C 1A4, Canada

**Keywords:** Stockholm Convention, long-range environmental transport, hazard, metrics, multimedia model, screening

## Abstract

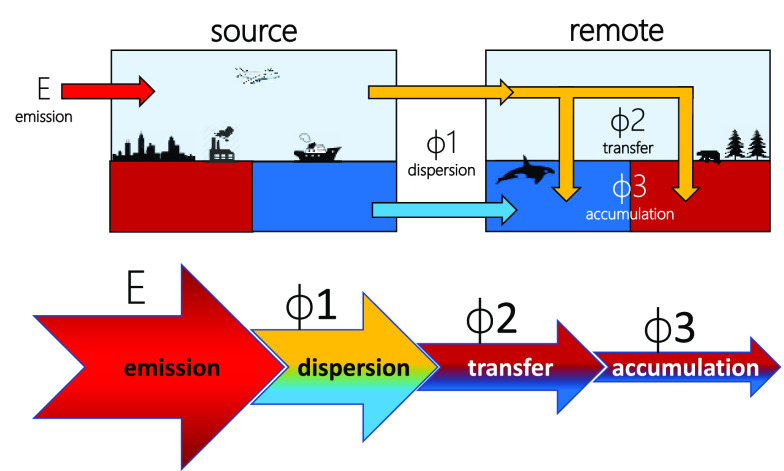

The assessment of long-range transport potential (LRTP) is enshrined in several frameworks for chemical regulation such as the Stockholm Convention. Screening for LRTP is commonly done with the OECD Pov and LRTP Screening Tool employing two metrics, characteristic travel distance (CTD) and transfer efficiency (TE). Here we introduce a set of three alternative metrics and implement them in the Tool’s model. Each metric is expressed as a fraction of the emissions in a source region. The three metrics quantify the extent to which the chemical (i) reaches a remote region (dispersion, ϕ1), (ii) is transferred to surface media in the remote region (transfer, ϕ2), and (iii) accumulates in these surface media (accumulation, ϕ3). In contrast to CTD and TE, the emissions fractions metrics can integrate transport via water and air, enabling comprehensive LRTP assessment. Furthermore, since there is a coherent relationship between the three metrics, the new approach provides quantitative mechanistic insight into different phenomena determining LRTP. Finally, the accumulation metric, ϕ3, allows assessment of LRTP in the context of the Stockholm Convention, where the ability of a chemical to elicit adverse effects in surface media is decisive. We conclude that the emission fractions approach has the potential to reduce the risk of false positives/negatives in LRTP assessments.

## Abstract 2

A new approach to LRTP assessment involving three related metrics seeks to overcome the shortcomings of existing methods while retaining conceptual simplicity.

## 1 Introduction

Concern related to long range atmospheric transport (LRAT) of pollution dates back 50 years^1^ and has led to international agreements such as the UNECE Convention on Long-Range Transboundary Air Pollution (CLRTAP)^2,3^ and the Stockholm Convention (SC) on Persistent Organic Pollutants (POPs).^4^ Over time, the regulatory interest has evolved toward a broader view of long-range transport (LRT), with long-range transport via water (LRWT) becoming an important consideration. The potential to undergo long-range transport (LRTP) to remote regions is a key hazard criterion to be met for an organic chemical to be listed under CLRTAP and the SC.^4^ In the SC, the requirement for listing is “the chemical is likely as a result of its long-range environmental transport to lead to significant adverse human health and/or environmental effects”, i.e., the chemical must not only be transported to remote regions, it must also accumulate in surface media there to an extent sufficient to cause harm.

Mathematical models play an important role in the scientific support of regulatory efforts, and a number of model-derived metrics has been developed for LRTP assessment,^5−10^ whereby transport- and target-oriented metrics are distinguished. The former address the potential of a chemical for widespread dispersal in air and/or water.^5^ Examples include the characteristic travel distance (CTD),^11−13^ the spatial range (SR),^14,15^ and the outflow ratio (OR).^7^ Because adverse effects of POPs arise from dietary uptake and transfer in food webs and rarely, if ever, from respiratory exposure, the SC considers the “transfer to a receiving environment in locations distant from the sources of its release” an integral part of LRT.^16^ Examples of target-focused metrics that seek to explicitly account for transfer of chemicals to surface media include the transfer efficiency (TE in %)^17,18^ and the Arctic contamination potential (ACP).^19^

A wide range of models with different levels of sophistication have been developed over the years to calculate these LRTP metrics.^5,7^ Following initiatives by the Organisation for Economic Co-operation and Development (OECD) and the United Nations Environment Programme (UNEP),^20^ an expert group was established in 2001 to provide guidance on how to use multimedia models in assessments of LRTP and overall persistence (*P*_OV_).^21^ A consensus model for LRTP and *P*_OV_ assessments, the OECD Overall Persistence and Long-Range Transport Potential Screening Tool (“the Tool”) was developed to support decision making for chemical management.^17^ The Tool calculates the CTD and the TE and it has found wide use in scientific research and regulatory practice, e.g., refs (22−25). For these calculations it employs a multimedia model which was deliberately designed to be as simple as possible, e.g., with respect to the number of compartments and the use of the steady-state assumption.

Transport- and target-oriented metrics are clearly related: only chemicals dispersed widely can be transferred to, and accumulate in, remote surface media. Curiously, no attempt has so far been made to establish metrics where that relationship is made explicit or quantified. Similarly, although the possibility for dispersal in air and water, and even the interaction of the two dispersal paths,^5,7^ is generally acknowledged, separate metrics for LRT in air and water are usually defined and rarely integrated. For example, there is no apparent way to combine CTDs in air and water to characterize overall LRT, and the TE, as implemented in the Tool, does not allow for the possibility that a chemical is transferred to the remote environment in water. Clearly, there is room to improve on the existing metrics for LRTP assessment.

The objectives of this study were to develop and introduce a coherent and integrated mechanistic approach to LRTP assessment that builds on a set of new transport- and target-oriented metrics that overcomes many of the limitations of the existing approaches. While these metrics should be intuitive and have an easily grasped meaning, one of them should explicitly assess a chemical’s potential for LRT in the sense of the SC. The point of departure was the desire to express quantitatively the relative extent to which a chemical can (i) reach remote regions, (ii) be transferred to surface media in remote regions, and (iii) accumulate in surface media in remote regions. Importantly, the relevant metrics should not be separate entities, but relate to, and complement, each other in a mechanistically meaningful manner. While the new metrics can be obtained with a variety of models of different levels of sophistication, a guiding principle during the design of the new approach was the need to have it implementable in very simple, steady-state models. Therefore, we introduce it here while relying on the fate model and parametrization in the OECD Tool.

## 2 Materials and Methods

### 2.1 OECD Tool Model Environment

**Figure 1:**
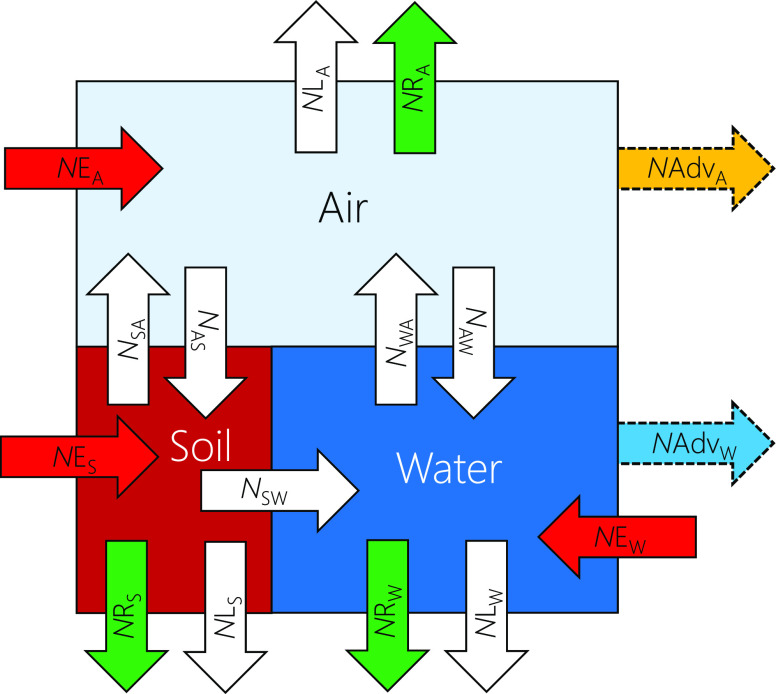
Compartments and processes of the level III steady state mass balance model in the Tool. A white arrow represents a transport flux, either occurring between air (A), soil (S), or water (W) within the model domain or from compartment X to the rest of the world (*N*L_X_). A red arrow represents an emission flux into compartment X (*N*E_X_). A green arrow represents a permanent loss rate by reaction in a compartment (*N*R_X_). The advective flux of chemical via air (*N*Adv_A_ in yellow) and water (*N*Adv_W_ in blue) are calculated from the results of the steady state mass balance as described in the text.

The Tool, which has been described in detail by Wegmann et al.,^17^ is a steady-state multimedia mass balance model, classified as a level III fugacity model.^26−28^ Its three compartments are parametrized to represent the global environment: the troposphere, the soil surface layer, and the seawater surface layer (Figure 1). Intermedia transport occurs by diffusion and advection, and degradation can occur within each compartment. Bulk degradation is assumed for water and soil, whereas degradation in air is restricted to the gas phase. While equilibrium is assumed within each compartment, chemicals need not be in equilibrium between them.^17^ Consequently, the predicted chemical distribution within the model environment and the LRTP metrics depend on the mode of emissions. The Tool makes predictions for three individual emission scenarios, which are 100% emissions to air, water, or soil.

Whereas the Tool describes a closed world, Wegmann et al. apply a scheme to nevertheless quantify the advective outflow of chemical in air (*N*Adv_A_), which is required for the calculation of the TE.^17^ They assume that the wind in the Tool, which has a speed of 4 m/s or 14400 m/h, is blowing across a hypothetical area *A*adv_A_. This allows for an estimate of the amount of chemical which leaves the model region in air, without this flux becoming a part of the model’s mass balance equations. We have adopted a similar strategy to calculate outflow of chemical in water. The rationale and equations used to calculate CTD and TE in the Tool are included in Section S1.

Most process descriptions and many of the key environmental input parameters in the Tool trace their origin back to pioneering work by Mackay and co-workers.^27^ While this does not imply that there might not be a need to reassess some of them, we chose to largely rely on the existing parametrization as this allowed us to more readily explore how our model predictions align with the Tool. However, as the assumption of constant drizzle in the Tool underestimates the potential for LRAT of both highly water-soluble chemicals and chemicals sorbed to particles during periods without precipitation^29^ which could lead to false negative categorizations, we included a parametrization of intermittent precipitation in the Tool^30^ (see Section S2).

### 2.2 Metrics

**Figure 2:**
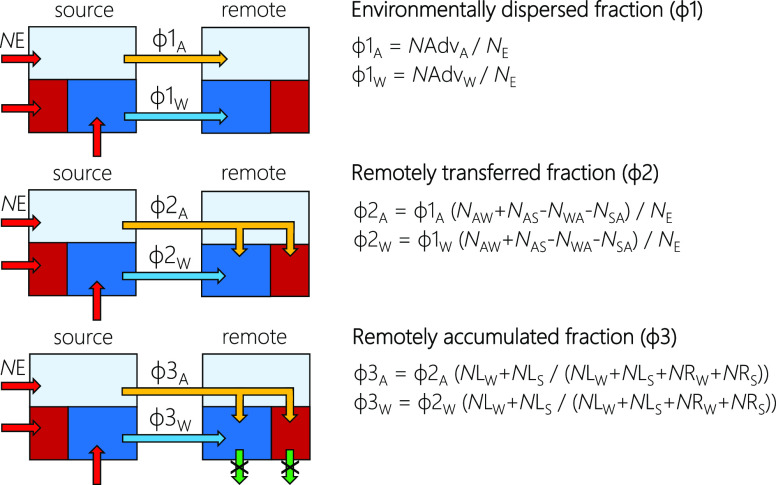
Representation of the three metrics in graphical terms. By only displaying elements crucial to the definition of the emission fractions, this model representation is simplified. For example, intermedia transfer in the source region and the reversibility of air surface exchange are not shown. See Figure 1 for all considered processes and an explanation of the colored arrows.

The emission fractions approach to LRTP assessment consists of a set of three coherent metrics. The environmentally dispersed fraction (ϕ1) quantifies the relative extent to which a chemical can reach remote regions. The remotely transferred fraction (ϕ2) expresses to what relative extent a chemical can reach surface media in remote regions. By accounting for degradative loss in surface media, the remotely accumulated fraction (ϕ3) assesses the fraction of chemical emissions accumulating in surface media of remote regions. Each metric is a fraction of the total amount emitted in the model environment as well as a fraction of the preceding metric. ϕ2 is a fraction of ϕ1 because only chemicals dispersed to a remote region can be transferred to the surface media there, and ϕ3 is a fraction of ϕ2, because only chemicals transferred to those media can accumulate in them. Figure 2 highlights how the three fractions can be conceptually represented in a steady-state model environment, along with the simplified equations. Please note that following an idea first proposed for the derivation of the TE in a simple steady-state mass balance model,^17^ we use the same model environment and parametrization for the source and the remote region, i.e., Figure 2 shows two environments that are represented by the same set of equations and input parameters. ϕ1 is a transport-oriented metric resembling the CTD. ϕ2 and ϕ3 are target-oriented metrics having similarities with the TE and the ACP, respectively.

#### 2.2.1 Environmentally Dispersed Fraction (ϕ1)

The relative potential for atmospheric dispersion is expressed as the fraction of emissions to all three media of the source region that is entering a remote region by air (A):
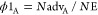
1where *N*adv_A_ (mol/h) is the outbound flux of chemical by advection into a hypothetical remote environment via the atmosphere, and *N*E (mol/h) is the emission flux. *N*adv_A_ is the product of the chemical concentration in air (mol/m^3^), the cross-sectional area across which atmospheric advection occurs (*A*adv_A_ in m^2^) and wind speed (m/h). The numerical value for *A*adv_A_ was selected in such a way that the chemicals with the highest atmospheric dispersion potential are assigned a ϕ1_A_ of 1 (i.e., *N*adv_A_ = *N*E). The maximum occurs for highly volatile chemicals emitted entirely into air (ϕ1_A_) which neither react in air nor net-deposit from the atmosphere to surface media. Using partitioning properties of log *K*_OA_ of 2 and log *K*_AW_ of 4 for this inert “flyer”, an area of 2.27 × 10^9^ m^2^ causes ϕ1_A_ to adopt a value of 1. This area is used for calculating ϕ1_A_ for any other chemical of interest.

The relative potential of a chemical to undergo environmental dispersion by water (ϕ1_W_) can be expressed using a similar equation:
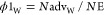
2where *N*adv_W_ (mol/h) is the outbound flux of chemical by advection into a hypothetical remote environment via water. Here we use the existing water flow velocity in the Tool (0.02 m/sec or 72 m/h). The cross-sectional area for advective outflow via water *A*adv_W_ was derived from *A*adv_A_ using
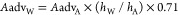
3where *h*_W_ and *h*_A_ are the heights of the water (100 m) and air compartment (6000 m) in the Tool, respectively, while 0.71 is the fraction of the global surface area covered by ocean. This results in an *A*adv_W_ of 2.68 × 10^7^ m^2^. This implies that a chemical would need to have a concentration in water 4.23 orders of magnitude higher than in air for air and water advection to be of similar importance.

Finally, the relative potential for a chemical to undergo environmental dispersion is calculated as
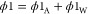
4

#### 2.2.2 Remotely Transferred Fraction (ϕ2)

ϕ2 expresses the relative extent to which a chemical is (net) transferred to surface compartments following environmental dispersion to a remote region. The relative extent to which a chemical can be transferred from air (A) to surface compartments (S = soil, W = water) following atmospheric dispersion (LRAT) to a remote region is calculated using
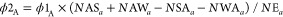
5Note that the fluxes (*N*-values) in eq 5 refer to model results for a scenario with emissions to air only, irrespective of what the mode of emission to the source environment had been. The equation for transfer to both surface media after dispersion in water (ϕ2_W_) is
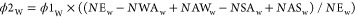
6

The fluxes in eq 6 always refer to a model scenario with 100% emissions to water (*N*E_W_) as only long-range transport with water (LRWT) into the remote region is being targeted.

The relative potential for a chemical to reach remote surface compartments following environmental dispersion is summarized as
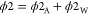
7

#### 2.2.3 Remotely Accumulated Fraction (ϕ3)

Accumulation in both surface compartments following atmospheric dispersion and net atmospheric deposition within the remote region (ϕ3_A_) is calculated using
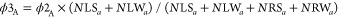
8where *N*LS_a_ and *N*LW_a_ describe soil burial and transfer to the deep sea, respectively, and *N*RS_a_ and *N*RW_a_ represent reaction in soil and water, respectively. Eq 8 expresses the fraction of deposited chemical that is retained in the respective medium (soil or water) but transferred to deeper layers. In this manner, accumulation tendency can be estimated using a steady state model. ϕ3_A_ is equal to ϕ2_A_ for persistent chemicals but is much smaller for chemicals that readily degrade. ϕ3_A_ thus quantifies not only the extent to which a chemical can reach remote surface compartments but also the extent to which it persists there. Again, the fluxes in eq 8 refer to model results for a scenario with emissions to air only. Using similar reasoning for dispersion in seawater (except that it references results for a scenario with 100% emissions to water) yields
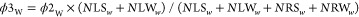
9

The relative potential for a chemical to accumulate in surface compartments following environmental dispersion is then summarized as
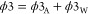
10

#### 2.2.4 Transfer and Accumulation in Soil or Water

The approach introduced above not only allows for calculations of emission fractions ϕ2 and ϕ3 where both surface compartments are lumped together. For more in-depth analyses, it is possible to quantify the relative potential for transfer to, and accumulation in, individual surface compartments. All equations employed for implementation in the Tool are included in Section S3.

Sometimes, the concern is not restricted to the LRT of the originally emitted chemical but comprises any persistent environmental transformation product(s). Building on earlier approaches advocating for “joint” assessment metrics,^31,32^ it would be straightforward to estimate the fraction of the emission of a chemical that is transferred, deposited, or accumulated in either its original form or as its persistent degradation product(s).

### 2.3 Visualizing Results

**Figure 3:**
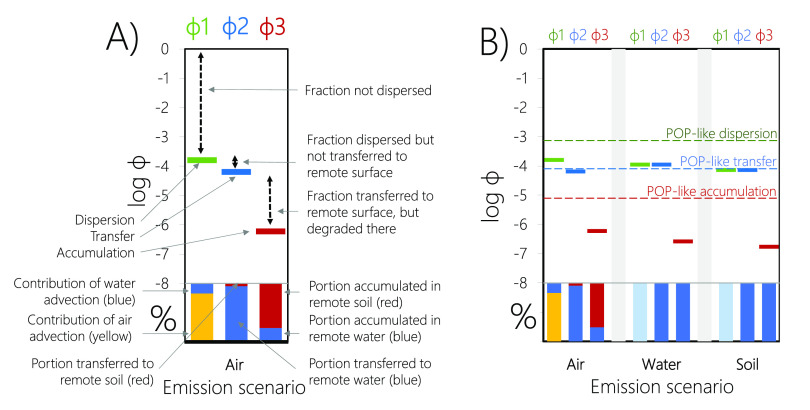
Visualization used to summarize the main results of the emissions fractions approach, using TCEP as an illustrative example. Panel A provides a description of the different elements of the figure using the example of the emission to air scenario. Panel B shows the complete visualization for all three emission scenarios and includes illustrative thresholds for POP-like LRTP behavior for each metric.

The coherency of the emission fractions approach, along with the additivity of the equations, allows for the display of the main results in a format that fosters a comprehensive understanding of the processes which lead to dispersion, transfer, and accumulation. Specifically, we propose to use graphs of the type shown in Figure 3 to summarize and display the results of the emissions fraction approach. Figure 3A uses TCEP [ethanol, 2-chloro-, phosphate (3:1)] as an illustrative example (Table S1) for the model scenario with 100% emissions to air. The three emissions fractions are designated by three colored markers in the upper part of the graph: green for the environmentally dispersed fraction, blue for the remotely transferred fraction, and red for the remotely accumulated fraction. The very wide range of values for emissions fractions necessitates the use of a logarithmic scale with an upper bound of log_10_ ϕ of 0, i.e., a ϕ of 1. For TCEP emitted to air, ϕ1 equals 0.016% (log_10_ ϕ1 = −3.8), ϕ2 equals 0.006% (log_10_ ϕ2 = −4.2), and ϕ3 is 0.00006% (log_10_ ϕ3 = −6.2). The difference between the top of the graph and the position of the green marker corresponds to the fraction not dispersed, the difference between blue and green markers represents the fraction dispersed but not transferred to remote surface compartments, and the difference between red and blue markers is the fraction transferred but not accumulated. The stacked colored bars with a scale of 0 to 100% in the lower parts of Figure 3A,B provide additional information. Those shown below ϕ1 indicate the relative importance of air and water advection to the chemical’s dispersal, i.e., LRAT (yellow) and LRWT (blue). The bars placed below ϕ2 and ϕ3 designate the relative portions transferred to, and accumulated in, the soil (red) and water (blue) of the remote region, respectively.

The three panels of Figure 3B further allow for a comparison of the results for the three modes of emissions. For illustration and to offer context for the numerical results for each of the three metrics, Figure 3B includes lines designating thresholds that separate chemicals that have POP-like LRTP from those that do not. Following Wegmann et al.,^17^ we defined these lines based on the lowest ϕ-value obtained for a subset of 14 discrete chemicals belonging to the initial “dirty dozen” POPs. These compounds have well characterized physical-chemical properties and fulfill the SC’s half-life criteria for persistence (see Table S2). We emphasize that what constitutes LRT is not primarily a scientific question but depends on the regulatory context. By using SC POPs to define the lines, they designate thresholds for global scale LRT. In a different regulatory context, different thresholds may apply, which could be defined on an expanded set of compounds that are deemed to satisfy the criterion of LRT on a smaller scale.

## 3 Results and Discussion

### 3.1 Application 1: Exploring LRTP Behavior of a Chemical

**Figure 4:**
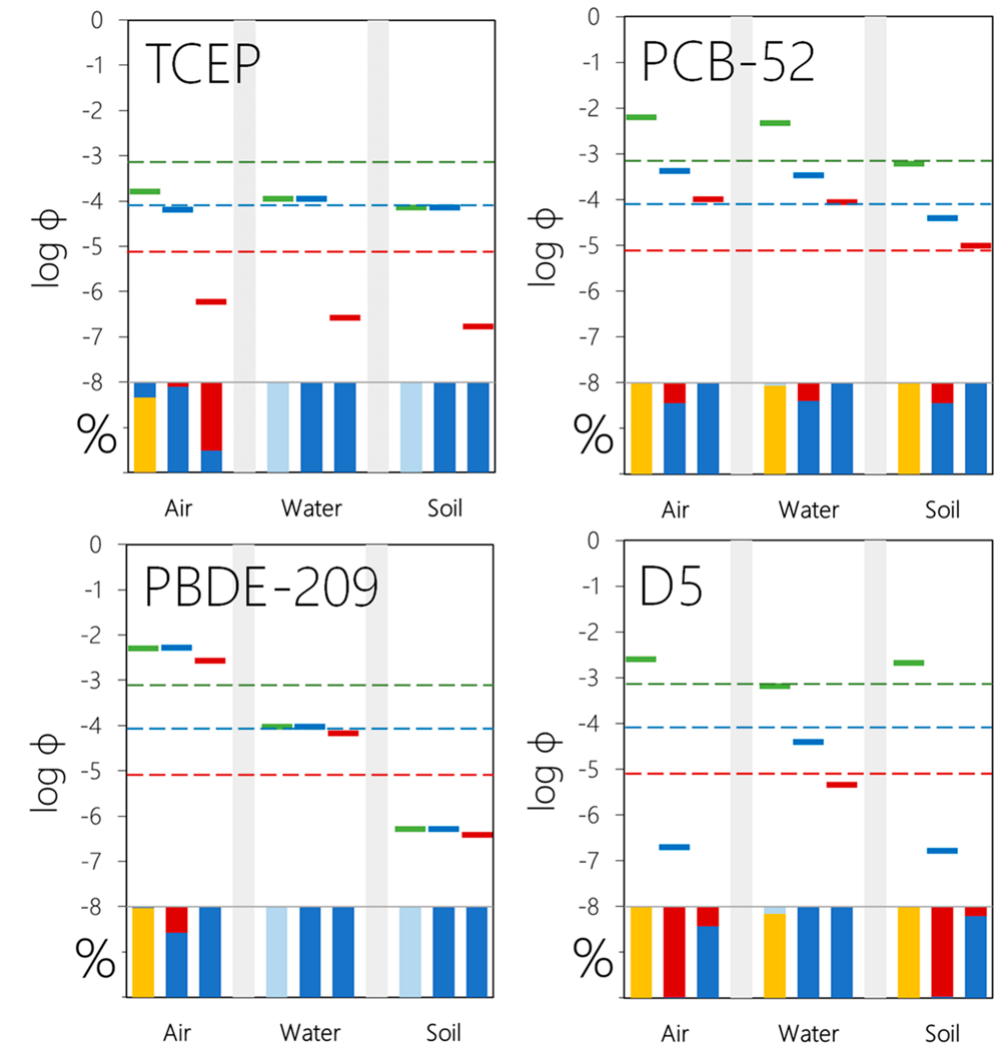
Results for a selection of chemicals with highly different LRTP behavior for three emission scenarios (see Figure 3 for explanation of legends).

The Tool was used to calculate the emission fractions for four selected chemicals with highly divergent LRTP behavior using three modes of emission (Figure 4). TCEP (log *K*_AW_ = −7.5, log *K*_OW_ = 1.7) is a highly water-soluble chemical (or “swimmer”). PCB-52 (log *K*_AW_ = −1.96, log *K*_OW_ = 6.26) is a semivolatile organic contaminant (SVOC or “multi-hopper”). PBDE-209 (log *K*_AW_ = −6.6, log *K*_OW_ = 8.7) is an involatile chemical (or “single-hopper”) with a log *K*_OA_ of 15.3. Whereas PBDE-209 is predicted to be completely sorbed to particles in air, D5 (log *K*_AW_ = 3.16, log *K*_OW_ of 6.78) is a highly volatile chemical (or “flyer”) which occurs as a vapor in the atmosphere.

#### 3.1.1 TCEP

If this chemical is emitted to water or soil, ϕ1 equals ϕ2, i.e., all of the dispersed chemical is deposited to surface media in the remote region (Figure 4). From the stacked bars in the lower part of the graph, we see that this is because TCEP is predicted to reach the remote region by advection in water (>99.9%). This result supports the study by Sühring et al.^22^ who recently suggested modifications to the Tool to expand its utility for chemicals which are more prone to undergo LRWT.^22^ Only if TCEP is emitted to air is there a difference between ϕ1 (dispersion) and ϕ2 (transfer), whereby ∼83% of the outflow from the source region is predicted to occur in air and the remainder in water. The latter contribution reflects the potential of TCEP to undergo wet deposition in the source region, followed by LRWT into the remote region. Notably, only a very small fraction of deposited TCEP accumulates in the remote region (ϕ3).

#### 3.1.2 PCB-52

The potential of PCB-52 for dispersion is attributed to LRAT irrespective of the mode of emission. As a persistent SVOC, PCB-52 is transferred quite readily between the three compartments in comparison to the other three chemicals. In particular, PCB-52 volatilizes from surface media to which it has been emitted in source regions, allowing for LRAT (ϕ1), followed by transfer (ϕ2) and accumulation (ϕ3). This explains why the relative distribution in terms of modes of transport, transfer, and accumulation show very minor differences across emission scenarios (stacked bars in Figure 4).

#### 3.1.3 PBDE-209

If an involatile chemical like PBDE-209 is emitted to media other than air, then the mode of dispersion into the remote region, transfer, and accumulation will all be associated with the water compartment (i.e., ϕ1 = ϕ1_W_, ϕ2 = ϕ2_W_, and ϕ3 = ϕ3_W_), see stacked bars in Figure 4. If PBDE-209 is emitted to air, it has a high ϕ1 (0.5%) with only a minor fraction being dispersed in water (1.3%). ϕ2 equals ϕ1 for PBDE-209 because the fraction dispersed in air is predicted to deposit on surface media in the remote region (ϕ2_A_ = ϕ1_A_: eq 5), whereas the fraction dispersed in water is identical to the fraction which enters water in the remote region (ϕ2_W_ = ϕ1_W_: eq 6). The relative portion transferred from air to each of the surface compartments of an involatile chemical as PBDE-209 is therefore by and large a reflection of the area fractions for soil and water in the model (71% water, 29% soil) as seen from the stacked bar in Figure 4. Given the default model assumption in the Tool, namely that particle-sorbed chemicals are persistent in air, the LRAT of PBDE-209 (as well as any other involatile chemical) is identical to the LRAT of the atmospheric particles. Reactions on particle surfaces have been shown to markedly reduce the atmospheric residence times of some involatile organic contaminants.^33^ Given the large number of chemicals in commerce which fall into this category,^23,34,35^ this model assumption may lead to a significant risk for false positive LRATP categorizations. Opportunities to improve descriptions of processes that affect the LRAT behavior of involatile chemicals should therefore be pursued in future work.

#### 3.1.4 D5

D5 is highly volatile and LRT is largely a result of LRAT, irrespective of mode of emissions (Figure 4, yellow bars). It has been shown to undergo LRAT where and/or when phototransformation is slow, such as at higher latitudes and/or during winter.^36^ D5 is predicted to have a very limited potential for atmospheric removal by deposition (ϕ2_A_), and the relative potential for D5 to accumulate in surface media as a result of atmospheric deposition is minuscule (log ϕ3 < −9.5). Because log *K*_AW_ of D5 is very high (3.16), its potential for transfer from air to water is even smaller than that for transfer from air to soil (stacked bars in Figure 4). The potential for environmental dispersion (ϕ1) is lowest if D5 is emitted to water where the potential for volatilization is mitigated by sorption of D5 to solids (log *K*_OW_ = 6.78). The latter emission scenario also leads to a trapping effect as D5 is more persistent in water than air. The highest potential for both transfer (ϕ2) and accumulation (ϕ3) is therefore predicted when D5 is emitted to water. We highlight the divergence of the LRTP categorization of D5 based on the CTD (or ϕ1) vs ϕ3 (or ϕ2). One could argue that judging D5 as having high LRTP in the sense of the SC based on its high CTD is a false positive decision because of its failure to accumulate in remote surface media to an extent sufficient to cause harm.

##### Impact of Mode of Emissions

Model scenarios involving 100% emissions into air usually represent the “worst-case” when it comes to a chemical’s potential for environmental dispersion (ϕ1). This is the case for the four chemicals in Figure 4. However, as the emission fractions approach, unlike the CTD and TE, explicitly accounts for chemical outflow with water, 100% emissions to air may not necessarily be the scenario which also leads to the highest ϕ2 and ϕ3 as noted for D5. Interestingly, the potential of TCEP for dispersion (ϕ1) and accumulation in remote surface media (ϕ3) are highest if emitted to air, whereas the potential for transfer to remote surface compartments (ϕ2) is highest if emitted to water. For ϕ1 and ϕ3 in the air emission scenario, a significant portion of the transport to the remote region occurs via water (17% and 24%, respectively, Figure 4). These observations illustrate the need to both account for LRWT^19,22^ and consider emissions to air in LRTP assessment of swimmers.

### 3.2 Application 2: Comparing the LRTP Behavior of Different Chemicals with Each Other and with Threshold Values

**Figure 5:**
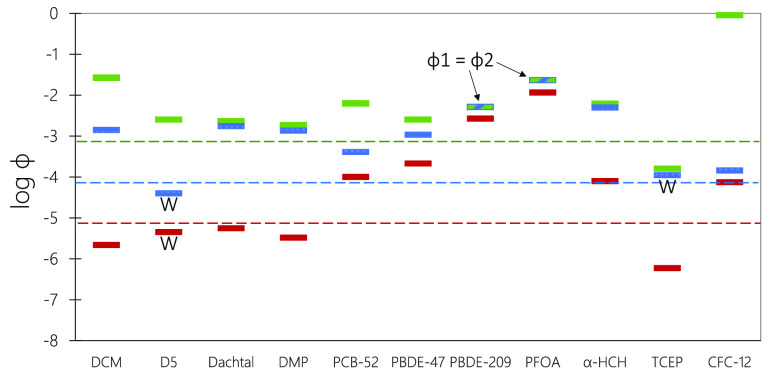
Predicted maximum values from the three emission scenarios for each of the three metrics for eleven selected chemicals (see Table S1 for explanation of acronyms). The dotted lines represent illustrative thresholds for potential POP-like dispersion (ϕ1, green), transfer (ϕ2, blue), and accumulation (ϕ3, red). The letter “W” indicates that the maximum value is predicted for the model scenario with 100% emissions to water. For all other values, the maximum values occur when the chemical is emitted to air, or they are identical across these two emission scenarios.

When using the emission fractions approach to compare several chemicals with respect to their LRTP, it may be advisable to focus on selected results, such as the maximum values for ϕ1, ϕ2, and ϕ3 obtained for any of the three emission scenarios. Figure 5 shows those values for eleven selected chemicals, together with the illustrative thresholds for POP-like LRTP behavior. Standard figures for the seven additional chemicals which were not included in Figure 4 are shown in Figure S2.

On the basis of the worst-case emission scenario, ten out of eleven chemicals are above the threshold for POP-like dispersion (ϕ1), ten are also above the threshold for POP-like transfer (ϕ2) although not the same chemicals, while six remain above the threshold for POP-like accumulation (ϕ3). With ϕ3 values above the red threshold line, Figure 5 attributes POP-accumulation behavior to chemicals listed under the SC (PCB-52, PBDE-47, PBDE-209, PFOA, and α-HCH), whereas those which are not (DCM, D5, Dacthal, DMP, and TCEP) have a ϕ3 below that line. An exception is CFC-12, which is assigned POP-like accumulation even though it is not listed under the SC. Figure 5 suggests that ϕ3 has the potential to identify chemicals for potential listing under the SC. It also highlights that neither a metric for dispersion (ϕ1 or CTD) nor transfer (ϕ2 or TE) is sufficient to do that. For example, an LRTP assessment based solely on a transport-oriented metric such as ϕ1 would indicate POP-like dispersion for D5, although its transfer to remote surface media is very limited. Similarly, a CTD and a TE-based assessment will assign LRTP above POP-like thresholds to many chemicals that are too degradable to accumulate in remote surface compartments. Overall, the results in Figure 5 are consistent with expectations based on existing knowledge and illustrate that the emission fractions approach has the power to differentiate between different kinds of LRTP behavior across a diverse set of chemicals.

### 3.3 Comparison of the Approach with Earlier Metrics

The choice of metrics has consequences for the outcome of an LRTP assessment. In Table 1, we have listed the main advantages and disadvantages of the existing and alternative metrics as implemented in the Tool with emphasis on risks for false positives/negatives, decision making contexts, and research needs.

**Table 1:** Advantages and Disadvantages of the Existing and Alternative Metrics

	OECD tool metrics	emissions fractions approach
transport-oriented metrics (CTD vs ϕ1)	same mechanistic information	
	has intuitive dimension	has intuitive meaning
	requires definition of wind speed	requires definition of wind speed and cross-sectional area
	combined assessment of LRAT and LRWT not possible	LRAT and LRWT are additive
transfer-oriented metrics (TE vs ϕ2)	gross deposition (for some chemicals TE > 100%)	net deposition
	includes transfer to remote region via air	includes transfer to remote region via air and water
target-oriented metrics (ϕ3)	not included	allows distinction between transfer to versus accumulation in remote surface media
combined metrics	not coherent	coherent and multiplicative, allowing quantitative comparison of different LRTP metrics

#### 3.3.1 ϕ1 versus CTD

CTD describes the potential for transport in the mobile media with simultaneous exchange with other media and predicts the distance from a point source at which the concentration of a chemical has been reduced to ∼37%. ϕ1_A_ is equal to CTD/CTD_max_ (where CTD_max_ is the CTD for an inert chemical), and hence ϕ1_A_ and CTD contain the same mechanistic information. CTD has dimensions of length. While a distance traveled by a chemical is intuitive and easily understood, the predicted distances should not be confused with actual transport distances in the real world. CTDs are not readily amenable to evaluation by higher-tier models that offer a more realistic representation of the environment. A metric which references emissions and estimates a fraction leaving a source region (ϕ1) aligns better with spatially resolved models predicting dispersion. For example, ϕ1 mirrors the outflow ratio (OR) which predicts the export out of a model domain as a result of advection.^7^ However, the major disadvantage of the CTD is that it is not able to express the combined dispersion in air and water. This is unlike ϕ1 which predicts the relative potential for dispersion in air, water, and both media combined. As the transport of chemical by either medium to a remote region should merit attention in the context of LRTP, we see this as a significant advantage.

#### 3.3.2 ϕ2 versus TE

The aim of both ϕ2 and TE is to quantify the relative potential of chemicals for transfer to surface media in remote regions. TE was originally introduced as a metric to identify chemicals which are prone to undergo gross atmospheric deposition to the Great Lakes following LRAT, using the spatially resolved BETR North America model.^18^ TE as implemented in the Tool is also restricted to consideration of gross atmospheric inputs. As recognized by Wegmann et al.,^17^ values for TE could therefore exceed 100% for chemicals undergoing repeated air-surface exchange (see also Figure S3). TE also does not account for transfer with seawater to the Tool’s remote region. This leads to a significant risk for false negatives for chemicals which are dispersed in water (LRWT) and therefore also in both air and water combined (LRT). ϕ2_A_ quantifies the net atmospheric deposition which is more relevant for LRTP assessments of SVOCs.^11,14,37,38^ ϕ2 furthermore accounts for transfer to surface compartments in remote regions as a result of both LRAT and LRWT (eq 7). The choice of the transfer-oriented metric has implications for ranking chemicals according to LRTP. Two examples demonstrate the shortcomings of the TE relative to ϕ2. PFOA has a low TE (ranked no. 9 out of the 11 chemicals in Figure 5; data not shown) but a high ϕ2 (ranked no. 1) because the latter metric correctly accounts for the considerable potential of PFOA for LRWT.^39^ By being based on the gross atmospheric deposition flux, the TE for CFC-12 is a nonintuitive 975%, whereas the ϕ2 for CFC-12 is 1.4 × 10^–04^, which correctly indicates that most of this chemical remains airborne and only a small fraction is transferred to surface media.

#### 3.3.3 ϕ3 versus ϕ2 and TE

Existing LRTP metrics have been classified as being either transport- or target-oriented. Here we deliberately distinguish between metrics quantifying transfer to (ϕ2), and accumulation (ϕ3) in, the remote region, and we think that this needs to be reflected in an expanded classification system of transport-, transfer-, and target-oriented metrics. While TE was introduced as a target-oriented metric, we believe this metric is better classified as a transfer-oriented metric along with ϕ2. ϕ3 provides an assessment of LRTP in the context of listing chemicals in the SC by offering an estimate of a chemical’s potential to be retained in the surface compartment(s) within a remote region, where the ability of a chemical to elicit adverse effects in surface media is decisive.

### 3.4 Strength and Limitations of the Emissions Fraction Approach

We used here the OECD Tool to introduce and illustrate the new system of LRT metrics because this widely accepted consensus model constitutes the current state-of-the-art in regulatory LRT assessment, as is for example evident in its frequent use in nominations of chemicals for listing in the SC. This should not be interpreted as an uncritical endorsement of this simple model and its current parametrization or as suggesting that there is no role for more complex models to play when assessing LRT. In fact, the emissions fraction approach to LRT assessment is not tied to a particular model but could be implemented with a variety of models of different spatial, temporal, and process resolution.

We have shown that the emission fractions approach introduced herein offers several advantages over the metrics currently implemented in the Tool. We therefore recommend that screening models such as the Tool include the emission fractions approach for coherent, transparent, and more reliable LRTP assessments. A clear advantage of the emission fractions approach in comparison to CTD and TE is that it helps identify and even quantify the more influential processes affecting LRT. We believe this aspect is important because there are some obvious, albeit deliberate, limitations to what a simple screening model as the Tool can do, such as accounting for temporal and spatial variability. It is thus important that simple screening models provide mechanistic insight to help guide higher-tier LRTP assessment. In future work, we will therefore explore how the emission fractions approach can be applied to gain more detailed insight into the pathways of transfer to, and accumulation in, individual surface compartments in remote regions.

## Supporting Information Available

Additional model equations, input parameters, and results (PDF)
