# Supplementary material for: The Emissions Fractions Approach to Assessing the Long-Range Transport Potential of Organic Chemicals
Source: Environ Sci Technol. 2022 Aug 11;56(17):11983–90. doi: 10.1021/acs.est.2c03047 (PMC9454247; doi:10.1021/acs.est.2c03047)
Supplement: Supplementary file 1 — es2c03047_si_001.pdf [file es2c03047_si_001.pdf]

Supporting Information

13 Pages, 2 Tables, 3 Figures

SUPPORTING INFORMATION

The emissions fractions approach to assessing the long-range transport potential of organic chemicals

Knut Breivik [1,2], Michael S. McLachlan [3] and Frank Wania [4]

[1] Norwegian Institute for Air Research, P.O. Box 100, NO-2027 Kjeller, Norway
[2] Department of Chemistry, University of Oslo, P.O. Box 1033, NO-0315 Oslo, Norway
[3] Department of Environmental Science, Stockholm University, SE-106 91 Stockholm, Sweden
[4] Department of Physical and Environmental Sciences, University of Toronto Scarborough, 1265 Military Trail, Toronto, Ontario, M1C 1A4, Canada

Contents

S1. Existing LRTP metrics calculated by The Tool S2
S1.1 Characteristic Travel Distance (CTD) S2
S1.2 Transfer Efficiency (TE) S2
S2. Intermittent precipitation S3
Figure S1 – Intermittent precipitation. S4
S3. Equations used to target individual compartments S4
S3.1 Transfer (ϕ2) S4
S3.2 Accumulation (ϕ3) S5
Table S1 – Selected chemicals S7
Table S2 – Acknowledged POPs S8
Figure S2 – Standard figures for 7 chemicals S9
Figure S3 - TE S11
References (Supporting Information) S12

S1. Existing LRTP metrics calculated by The Tool

S1.1 Characteristic Travel Distance (CTD)

In order to harmonize the nomenclate in the equations to follow we use subscript *i* to denote any of the three different possible single-media emission scenarios that are calculated by the Tool. These are 100% emissions to air, seawater, and soil, respectively (subscript *i* whereby *a* = air, *w* = seawater and *s* = soil). We furthermore use *N* to denote fluxes rather than *F* as used in Wegmann et al.[1] to align with the nomenclature used in the main manuscript. As the OECD Tool is a Level III model, the predicted environmental fate of chemicals and thereby also the predicted metrics depend on the mode-of-release.[2] The Tool thus calculates multiple predictions of CTD and TE (CTD_i in km and TE_i in %)[1], whereby CTD and TE, if reported without any indexes, refer to the highest value (worst-case scenario).

The general equation for CTD from Wegmann et al. is reproduced below, whereby CTD is calculated for any of the two mobile media (J = A or W). We note that *i* and J represent the same compartment in Equation S1:

$$CTD_{i,J} = M_{i,TOT} / NE_i * M_{i,J} / M_{i,TOT} * v_J = M_{i,J} * v_J / NE_i \quad \text{[Equation S1]}$$

In Equation 1, $M_{i,TOT}$ (moles) is the total chemical amount in the entire model environment for a specified emission scenario (*i*), whereas NE_i is the emission flux (mol/h) entering compartment *i*. The first term corresponds to the overall residence time (h) at steady-state. The second term represents the chemical fraction in the mobile compartment J. It is expressed as the ratio between the amount in the mobile compartment (J) to which the chemical was emitted $M_{i,J}$ divided by $M_{i,TOT}$. The last parameter is the transport velocity of the mobile compartment (v_J in km/h).

S1.2 Transfer Efficiency (TE)

The TE (%) represents the mass flux into one or more target surface compartments, divided by the emission mass flux in a source region with the aim to quantify how much chemical reaches a remote region. The TE was initially developed and applied to assess the relative potential of organic chemicals to undergo atmospheric transport and deposition to the Great Lakes.[3] The Tool is a non-spatially resolved model for which the world is described as a closed unit. In other words, there is no explicit source and target region, which represents a fundamental challenge for any attempt to predict LRTP metrics targeting the remote region. In order to calculate this metric Wegmann et al.[1] outline a conceptual approach whereby it is assumed that the Tool's model environment can be subdivided into two parts, consisting of a source and receptor region. These two regions are connected through a hypothetical plane. This approach relies on the presence of a hypothetical cross-sectional area across which transport from the source region and into the remote region takes place. In The Tool, the assumed cross-sectional area is set equal to the square root of the global surface area multiplied by the height of the atmosphere. We refer to Wegmann et al.[1] for details.

TE is described by Wegmann et al.[1] as the flux (in mol/h) into a specified compartment (X) in the remote region ($N'_{i,X}$) (the ′ symbol designates the remote region), divided by the emission flux ($N\mathrm{E}_i$, also in mol/h). Individual TEs (TE_i) are calculated separately for emissions to air, water and soil as the total atmospheric deposition mass flux to both surface compartments in the recipient region ($N\mathrm{D}_i$, divided by the emission flux ($N\mathrm{E}_i$). Note that $N\mathrm{D}_i$ is equal to $\mathrm{NAS}_i + \mathrm{NAW}_i$ as used in the main manuscript. As the The Tool is a linear model, it is argued that the ratio between the emission flux divided the atmospheric deposition flux in the source region ($N\mathrm{E}_i/N\mathrm{D}_i$) is identical to the ratio between the advective inflow flux into the remote region caused by LRT, divided by the atmospheric deposition mass flux within the remote region ($N\mathrm{adv}_i\,/N\mathrm{D}'_i$). This can be re-arranged as follows:

$N\mathrm{D}'_i = N\mathrm{D}_i * N\mathrm{D}_i / N\mathrm{E}_i$ [Equation S2]

Because TE_i is defined as $N\mathrm{D}'_i/N_{i,\mathrm{E}}$, substitution yields:

$\mathrm{TE}_i = 100\% * N\mathrm{D}_i * \mathrm{Nadv}_i / (N\mathrm{E}_i)^2$ [Equation S3]

S2. Intermittent precipitation

Figure S1 shows the predicted CTD in air for perfectly persistent chemicals under the current assumption of constant drizzle for the model scenario with 100% emissions to air. Under this scenario, the predicted CTD in air ranges from 8.9 km up to $1.35*10^5$ km across the chemical partitioning space. It is well established that the "constant drizzle" assumption underestimates LRAT of chemicals which are prone to wet deposition[4, 5]. Given the focus on LRT in this study, we therefore modified the code of The Tool account for intermittent precipitation as earlier implemented in the BETR-Global model.[6] Following MacLeod et al., we assumed the duration of dry and wet periods to be 12 and 120 hours, respectively.[7] This modification caused the CTD of a perfectly persistent chemical which is readily prone to removal from air by rain dissolution (log K_{AW} = -8, log K_{OA} of 2) to increase by more than an order of magnitude. For hypothetical inert chemicals which are readily removed from the atmosphere by wet and dry particle deposition (log K_{OA} > ~10), the CTD increased by a factor of ~2.5. The introduction of intermittent precipitation did not affect the predicted CTD for inert chemicals with a log K_{AW} > -5 and a log K_{OA} < 8. All results presented in this study account for intermittent precipitation.

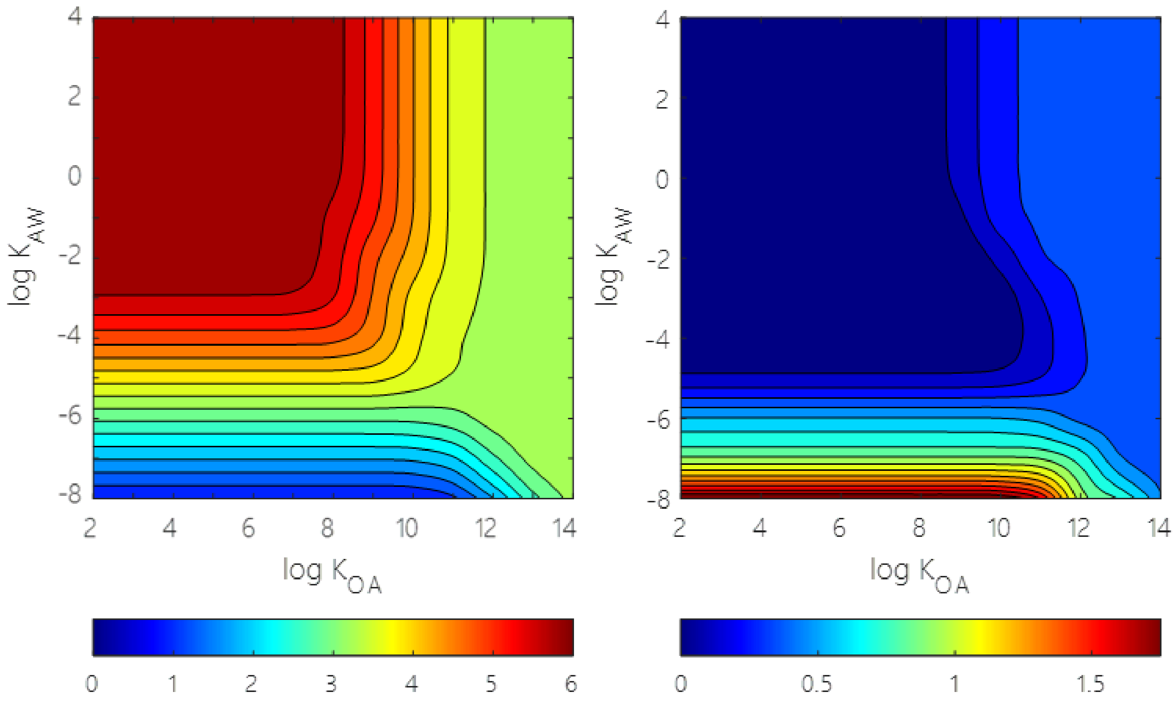

Figure S1 – Intermittent precipitation. Left panel: Predicted log CTD in air for perfectly persistent chemicals under the assumption of constant drizzle in The Tool. Right panel: Logarithmic ratio of predicted CTD in air under the intermittent precipitation scenario, divided by CTD in air under the assumption of constant drizzle (perfectly persistent chemicals). Results apply to the model scenario with 100% emissions to air.

S3. Equations used to target individual compartments

In the following equations, the capital letter which follows ϕ designates the surface compartment, which is either soil (S) or water (W). The subscript *i* in italics now defines the mode of the emission scenario ($_{i\,=\,\text{a, s, w}}$), whereas the last subscript in capital letter identifies the mode of dispersion (A = air, W = water, T = total).

S3.1 Transfer (ϕ2)

The relative extent to which a chemical can be transferred from air to soil or water following atmospheric dispersion (LRAT) to a remote region is calculated using:

$\phi 2S_{i,A} = \phi 1_{i,A} * (NAS_a - NSA_a - NSW_a) / NE_a$ [Equation S4]

$\phi 2W_{i,A} = \phi 1_{i,A} * (NAW_a - NWA_a + NSW_a) / NE_a$ [Equation S5]

Here, NAS_a represents the gross atmospheric deposition flux from air to soil, NSA_a represents the reversible flux from soil to air, and NSW_a is the transfer from soil to water (all in mol/h). The term NAW_a - NWA_a represents the net atmospheric deposition flux from air to water. Whereas the atmospherically dispersed fraction ($\phi 1_{i,A}$) can be calculated for any mode of emission ($_{i = a, s, w}$), the fluxes in equations S4 and S5 always refer to a model scenario with 100% emissions to air (NE_a) as only LRAT into the remote region is being targeted. Hence, the relative potential for a chemical to reach both remote surface compartments via the atmosphere ($\phi 2_{i,A}$) can not only be derived using Equation 5 in the main manuscript, but it can also be calculated as:

$$\phi 2_{i,A} = \phi 2S_{i,A} + \phi 2W_{i,A} \qquad \text{[Equation S6]}$$

Using equivalent reasoning we can calculate the extent to which a chemical is transferred to soil and water after dispersion to a remote region with water.

$$\phi 2S_{i,W} = \phi 1_{i,W} * (NAS_w - NSA_w - NSW_w) / NE_w \qquad \text{[Equation S7]}$$

$$\phi 2W_{i,W} = \phi 1_{i,W} * ((NE_w - NWA_w + NAW_w + NSW_w) / NE_w) \qquad \text{[Equation S8]}$$

While the fraction dispersed with water ($\phi 1_{i,W}$) can again be calculated for any mode of emission ($_{i = a, s, w}$), the fluxes in equations S7 and S8 always refer to a model scenario with 100% emissions to water (NE_W) as only long range transport with water into the remote region is being targeted. The equation for transfer to both surface media after dispersion in water ($\phi 2_{i,W}$) can then, as an alternative to Equation 6, be calculated as:

$$\phi 2_{i,W} = \phi 2S_{i,W} + \phi 2W_{i,W} \qquad \text{[Equation S9]}$$

Finally, the relative extent to which a chemical may be transferred to either or both surface compartments by environmental dispersion via both mobile media combined (T) can then be summarized:

$$\phi 2S_{i,T} = \phi 2S_{i,A} + \phi 2S_{i,W} \qquad \text{[Equation S10]}$$

$$\phi 2W_{i,T} = \phi 2W_{i,A} + \phi 2W_{i,W} \qquad \text{[Equation S11]}$$

$$\phi 2_{i,T} = \phi 2S_{i,T} + \phi 2W_{i,T} \qquad \text{[Equation S12]}$$

Equation S12 here offers the alternative to Equation 7.

S3.2 Accumulation (ϕ3)

Accumulation in soil or water as result of atmospheric dispersion into the remote region, followed by net atmospheric deposition within the remote region is calculated using:

$\phi 3S_{i,A} = \phi 2S_{i,A} * (NLS_a / (NLS_a + NRS_a))$ [Equation S13]

$\phi 3W_{i,A} = \phi 2W_{i,A} * (NLW_a / (NLW_a + NRW_a))$ [Equation S14]

Accumulation in both surface compartments within the remote region ($\phi 3_{i,A}$) can, as an alternative to Equation 8, be calculated using:

$\phi 3_{i,A} = \phi 3S_{i,A} + \phi 3W_{i,A}$ [Equation S15]

Accumulation in soil or water as result of dispersion in water can be calculated in a similar fashion, except that it references results for the model scenario with 100% emissions to water:

$\phi 3S_{i,W} = \phi 2S_{i,W} * (NLS_w / (NLS_w + NRS_w))$ [Equation S16]

$\phi 3W_{i,W} = \phi 2W_{i,W} * (NLW_w / (NLW_w + NRW_w))$ [Equation S17]

As an alternative to Equation 9, the total accumulation as result of dispersion in water can then be calculated using:

$\phi 3_{i,W} = \phi 3S_{i,A} + \phi 3W_{i,W}$ [Equation S18]

The total accumulation in soil and water as consequence of both modes of dispersion can then be derived using Equations S19 and S20, respectively:

$\phi 3S_{i,T} = \phi 3S_{i,A} + \phi 3S_{i,W}$ [Equation S19]

$\phi 3W_{i,T} = \phi 3W_{i,A} + \phi 3W_{i,W}$ [Equation S20]

Finally, the total remotely accumulated fraction as consequence of environmental dispersion and transfer to both surface media can be summarized as:

$\phi 3_{i,T} = \phi 3S_{i,T} + \phi 3W_{i,T}$ [Equation S21]

Or by using Equation 10.

Table S1 – Selected chemicals Molecular weight, physical-chemical properties (25°C) and environmental degradation half-lives (25°C).

Chemical	MW (g/mol)	log K_{AW} (-)	log K_{OW} (-)	$t_{1/2}$ air (hours)	$t_{1/2}$ water (hours)	$t_{1/2}$ soil (hours)
DCM (dichloromethane)	84.93	-0.91 [A]	1.43 [B]	1,903 [C]	900 [D]	1,800 [D]
D5 (decamethylcyclopentasiloxane)	370.77	3.16 [E]	6.78 [F]	166 [G]	996 [H]	302 [I]
Dacthal (dimethyl tetrachloroterephthalate)	331.97	-4.96 [A]	4.35 [B]	582 [C]	1,440 [D]	2,880 [D]
DMP (dimethyl phthalate)	194.19	-5.67 [A]	1.33 [B]	447 [C]	360 [D]	720 [D]
PCB-52 (2,2′,5,5′-tetrachlorobiphenyl)	291.99	-1.96 [J]	6.26 [J]	435 [K]	10,000 [K]	17,000 [K]
PBDE-47 (2,2',4,4',5-pentabromodiphenyl ether)	485.5	-3.35 [L]	6.39 [L]	257 [C]	4,320 [D]	8,640 [D]
PBDE-209 (decabromodiphenyl ether)	959.2	-6.57 [M]	8.7 [L]	7,618 [C]	4,320 [D]	8,640 [D]
PFOA (perfluorooctanoic acid)	414.07	-9.77 [N]	2.47 [O]	604,400 [P]	604,400 [P]	604,400 [P]
α-HCH (α-hexachlorocyclohexane)	290.9	-3.53 [Q]	3.94 [Q]	1,843 [R]	8,760 [S]	2,190 [S]
TCEP (tris(2-chloroethyl) phosphate)	285.49	-7.5 [D]	1.7 [D]	12 [C]	1,440 [D]	2,880 [D]
CFC-12 (dichlorodifluoromethane)	120.9	1.15 [D]	2.16 [D]	641,800 [T]	641,800 [U]	641,800 [U]

[A] UFZ-LSER database [8, 9]
[B] UFZ-LSER database [8, 10]
[C] EPIWIN [11], calculated from overall OH rate constant
[D] EPIWIN [11]
[E] Xu and Kropscott [12]
[F] Back-calculated from a log K_{OC} = 6.3 from Panagopoulos and MacLeod (2018) and references therein [13], using a relationship by Seth et al (1999) [14]
[G] Calculated on the basis of Atkinson (1989)[15], using the OH-radical concentration in EPIWIN [11]
[H] Average of two values from Panagopoulos and MacLeod (2018) and references therein [13]
[I] Panagopoulos and MacLeod (2018) and references therein [13]
[J] Schenker et al. (2005) [16]
[K] Wania and Su (2004) [17]
[L] Wania and Dugani (2003) [18]
[M] Calculated from log K_{OA} and log K_{OW} reported by Wania and Dugani (2003) [18]
[N] Assumed value
[O] Back-calculated from a log K_{OC} reported by Armitage et al (2006) [19], using a relationship by Seth et al (1999) [14]
[P] Armitage et al (2006) [19]
[Q] Xiao et al (2004) [20]
[R] Brubaker and Hites (1998) [21] and EPIWIN [11]
[S] Breivik and Wania (2002) [22]
[T] Atkinson (1989) using the OH-radical concentration in EPIWIN [11]
[U] Assumed similar reaction half-life in surface media as in air.

Table S2 – Acknowledged POPs Molecular weight, physical-chemical properties (25°C) and environmental degradation half-lives (25°C) for 12 chemicals acknowledged as POPs under the Stockholm Convention. See Table S1 for the corresponding input data for PCB-52 and α-HCH.

	MW (g/mol)	log K_{AW} (-)	log K_{OW} (-)	$t_{1/2}$ air (hours)	$t_{1/2}$ water (hours)	$t_{1/2}$ soil (hours)
γ-HCH	290.9	-3.91 [A]	3.83 [A]	1,351 [B]	8,760 [C]	2,190 [C]
PCB-28	257.54	-1.93 [D]	5.66 [D]	233 [E]	5,500 [F]	10,000 [F]
PCB-101	326.43	-2.08 [D]	6.38 [D]	755 [E]	31,000 [F]	100,000 [F]
PCB-118	326.43	-2.36 [D]	6.65 [D]	856 [G]	31,000 [G]	100,000 [G]
PCB-138	360.88	-1.97 [D]	7.19 [D]	1,605 [G]	55,000 [G]	170,000 [G]
PCB-153	360.88	-2.13 [D]	6.86 [D]	1,605 [G]	55,000 [G]	550,000 [G]
PCB-180	395.32	-2.51 [D]	7.15 [D]	2,334 [E]	55,000 [F]	1,000,000 [F]
HCB	284.8	-3.91 [H]	3.83 [H]	15,190 [I]	4,320 [I]	8,640 [I]
pp-DDT	354.5	-3.34 [H]	6.39 [H]	75 [I]	4,320 [I]	8,640 [I]
pp-DDD	321	-3.70 [H]	6.33 [H]	59 [I]	4,320 [I]	8,640 [I]
cis-Chlordane	409.8	-2.63 [H]	6.20 [H]	51 [I]	4,320 [I]	4,320 [I]
Trans-chlordane	409.8	-2.56 [H]	6.27 [H]	51 [I]	4,320 [I]	4,320 [I]

[A] Xiao et al (2004)[20]
[B] Brubaker and Hites (1998)[21]
[C] Breivik et al (2002) [22]
[D] Schenker et al (2005)[16]
[E] Beyer et al (2003)[23]
[F] Wania and Daly (2002)[24]
[G] Wania and Su (2004)[17]
[H] Shen and Wania (2005)[25]
[I] EPIWIN [11]

Figure S2 – Standard figures for 7 chemicals Results for a selection of chemicals with highly different LRTP behavior by emission scenario (see Figure 3 for explanation of legends).

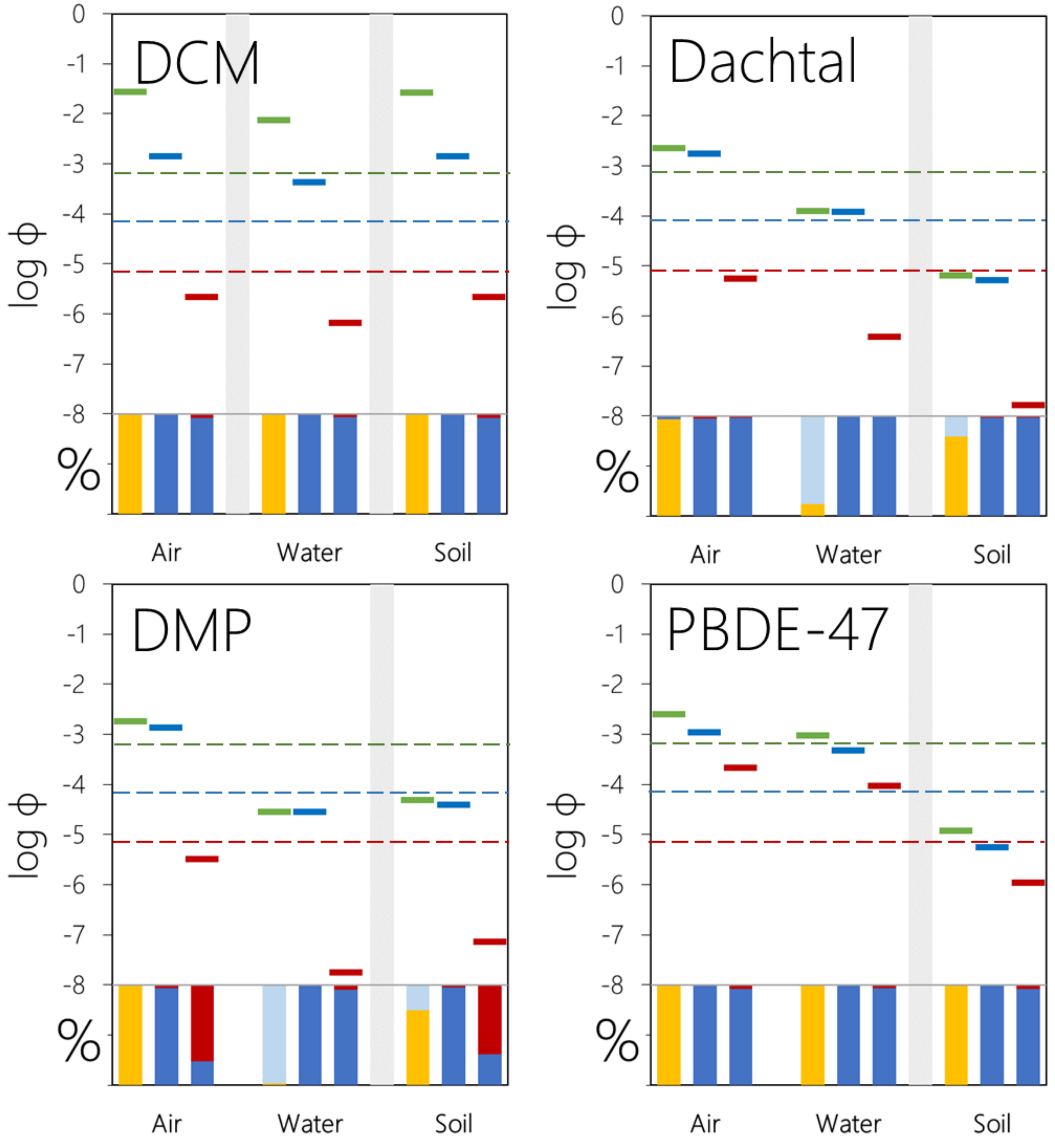

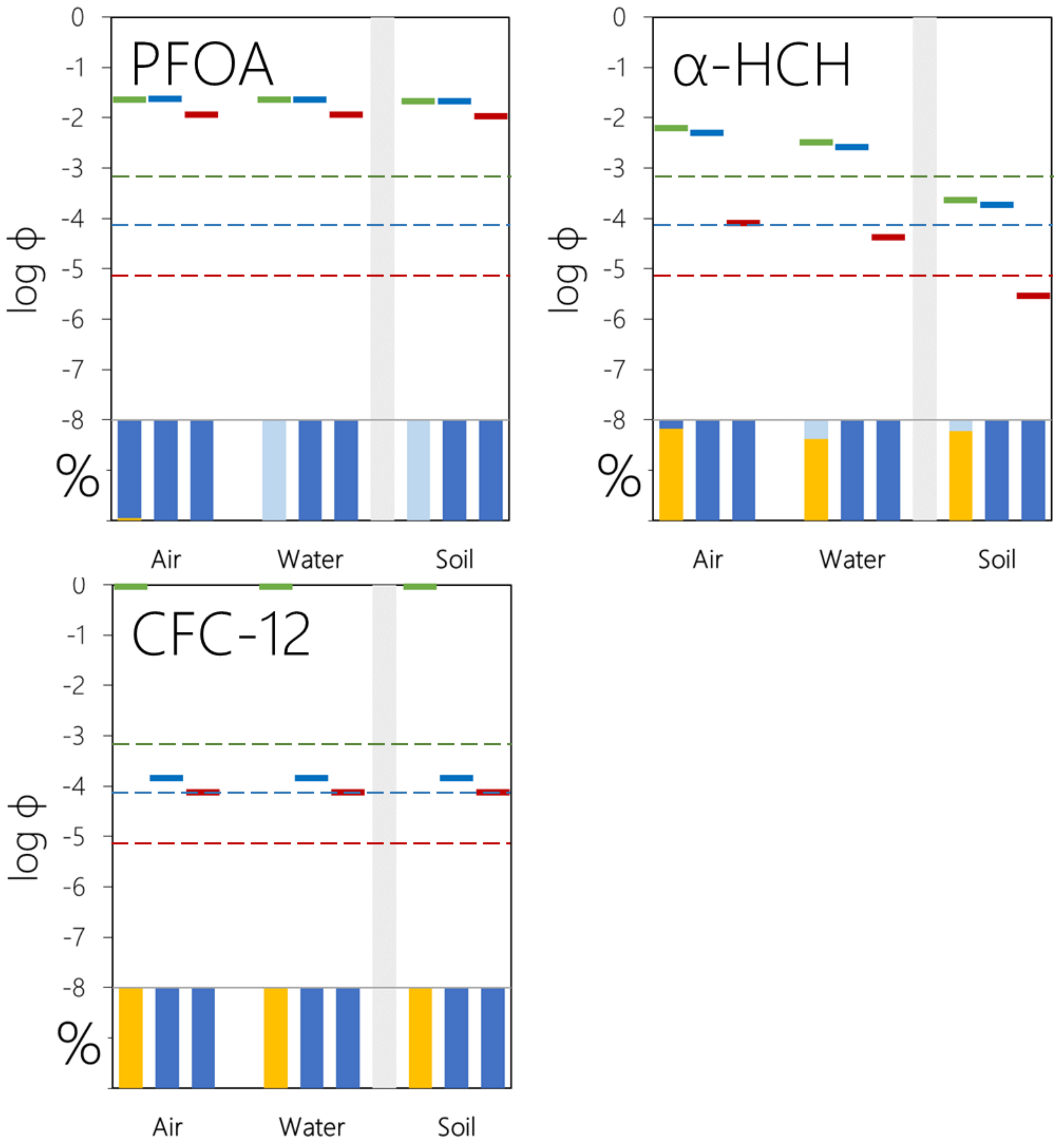
PFOA
α-HCH
CFC-12
log φ
%
0
-1
-2
-3
-4
-5
-6
-7
-8
Air
Water
Soil

Figure S3 - TE log TE (%) for perfectly persistent chemicals for the model scenario with 100% emissions to air.

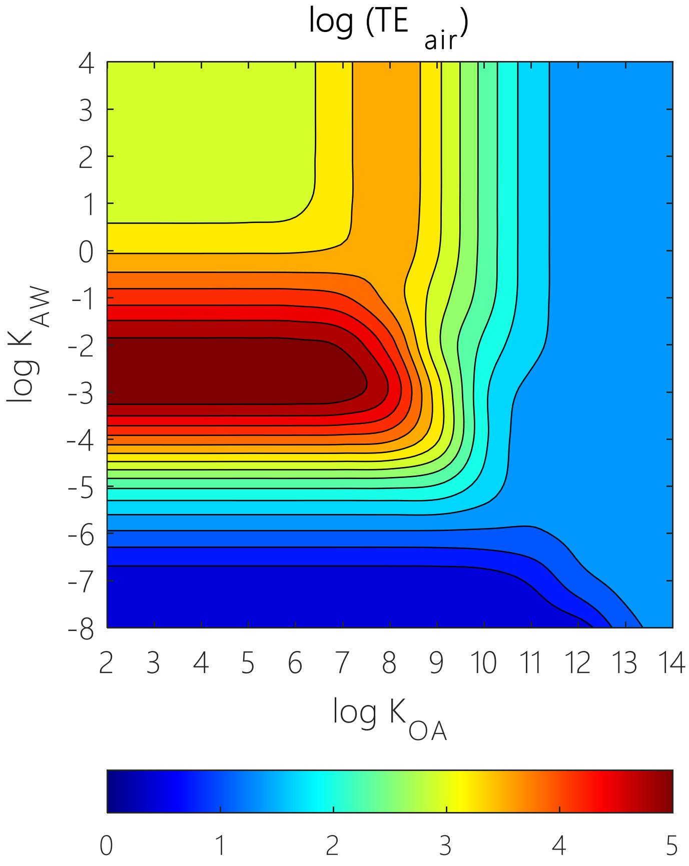

References (Supporting Information)

1. Wegmann, F.; Cavin, L.; MacLeod, M.; Scheringer, M.; Hungerbuhler, K., The OECD software tool for screening chemicals for persistence and long-range transport potential. *Environmental Modelling & Software* **2009,** *24*, (2), 228-237. doi:10.1016/j.envsoft.2008.06.014.
2. Mackay, D., *Multimedia Environmental Models: The Fugacity Approach*. 2 ed.; CRC Press, Boca Raton, FL: 2001; p 272.
3. MacLeod, M.; Mackay, D., Modeling transport and deposition of contaminants to ecosystems of concern: a case study for the Laurentian Great Lakes. *Environ. Pollut.* **2004,** *128*, (1-2), 241-250. doi:10.1016/j.envpol.2003.08.029.
4. Hertwich, E. G., Intermittent rainfall in dynamic multimedia fate modeling. *Environ. Sci. Technol.* **2001,** *35*, (5), 936-940. doi:10.1021/es000041v.
5. Jolliet, O.; Hauschild, M., Modeling the influence of intermittent rain events on long-term fate and transport of organic air pollutants. *Environ. Sci. Technol.* **2005,** *39*, (12), 4513-4522. doi:10.1021/es049913+.
6. MacLeod, M.; von Waldow, H.; Tay, P.; Armitage, J. M.; Wohrnschimmel, H.; Riley, W. J.; McKone, T. E.; Hungerbuhler, K., BETR global - A geographically-explicit global-scale multimedia contaminant fate model. *Environ. Pollut.* **2011,** *159*, (5), 1442-1445. doi:10.1016/j.envpol.2011.01.038.
7. MacLeod, M.; Scheringer, M.; Götz, C.; Hungerbühler, K.; Davidson, C. I.; Holsen, T. M., *Chapter 6. Deposition from the Atmosphere to Water and Soil with Aerosol Particles and Precipitation*. In: Thibodeaux, L.J.; Mackay, D. (Eds) Handbook of Chemical Mass Transport in the Environment. CRC Press: Boca Ration, FL, 2010.
8. Ulrich, N.; Endo, S.; Brown, T. N.; Watanabe, N.; Bronner, G.; Abraham, M. H.; Goss, K. U., UFZ-LSER database v 3.2 [Internet]. **2017**.
9. Goss, K. U., Predicting the equilibrium partitioning of organic compounds using just one linear solvation energy relationship (LSER). *Fluid Phase Equilibria* **2005,** *233*, (1), 19-22. doi:10.1016/j.fluid.2005.04.006.
10. Abraham, M. H.; Smith, R. E.; Luchtefeld, R.; Boorem, A. J.; Luo, R. S.; Acree, W. E., Prediction of Solubility of Drugs and Other Compounds in Organic Solvents. *Journal of Pharmaceutical Sciences* **2010,** *99*, (3), 1500-1515. doi:10.1002/jps.21922.
11. US-EPA *Estimation Programs Interface Suite™ for Microsoft® Windows, v 4.11*, United States Environmental Protection Agency: Washington, DC, USA, 2021.
12. Xu, S. H.; Kropscott, B., Evaluation of the three-phase equilibrium method for measuring temperature depedence of internally consistent partition coefficients (K-OW, K-OA, and K-AW) for volatile methylsiloxanes and trimethylsilanol. *Environmental Toxicology and Chemistry* **2014,** *33*, (12), 2702-2710. doi:10.1002/etc.2754.
13. Panagopoulos, D.; MacLeod, M., A critical assessment of the environmental fate of linear and cyclic volatile methylsiloxanes using multimedia fugacity models. *Environmental Science-Processes & Impacts* **2018,** *20*, (1), 183-194. doi:10.1039/c7em00524e.
14. Seth, R.; Mackay, D.; Muncke, J., Estimating the organic carbon partition coefficient and its variability for hydrophobic chemicals. *Environ. Sci. Technol.* **1999,** *33*, (14), 2390-2394.

15. Atkinson, R., Kinetics and Mechanisms of the Gas-Phase Reactions of the Hydroxyl Radical with Organic Compounds. *Journal of Physical and Chemical Reference Data* **1989,** *Monograph No. 1*.
16. Schenker, U.; MacLeod, M.; Scheringer, M.; Hungerbuhler, K., Improving data quality for environmental fate models: A least-squares adjustment procedure for harmonizing physicochemical properties of organic compounds. *Environ. Sci. Technol.* **2005,** *39*, (21), 8434-8441. doi:10.1021/es0502526.
17. Wania, F.; Su, Y. S., Quantifying the global fractionation of polychlorinated biphenyls. *Ambio* **2004,** *33*, (3), 161-168.
18. Wania, F.; Dugani, C. B., Assessing the long-range transport potential of polybrominated diphenyl ethers: A comparison of four multimedia models. *Environmental Toxicology and Chemistry* **2003,** *22*, (6), 1252-1261.
19. Armitage, J.; Cousins, I. T.; Buck, R. C.; Prevedouros, K.; Russell, M. H.; MacLeod, M.; Korzeniowski, S. H., Modeling global-scale fate and transport of perfluorooctanoate emitted from direct sources. *Environ. Sci. Technol.* **2006,** *40*, (22), 6969-6975. doi:10.1021/es0614870.
20. Xiao, H.; Li, N. Q.; Wania, F., Compilation, evaluation, and selection of physical-chemical property data for alpha-, beta-, and gamma-hexachlorocyclohexane. *Journal of Chemical and Engineering Data* **2004,** *49*, (2), 173-185. doi:10.1021/je034214i.
21. Brubaker, W. W.; Hites, R. A., OH reaction kinetics of gas-phase alpha- and gamma-hexachlorocyclohexane and hexachlorobenzene. *Environ. Sci. Technol.* **1998,** *32*, (6), 766-769.
22. Breivik, K.; Wania, F., Evaluating a model of the historical behavior of two hexachlorocyclohexanes in the Baltic sea environment. *Environ. Sci. Technol.* **2002,** *36*, (5), 1014-1023. doi:10.1021/es001971h.
23. Beyer, A.; Wania, F.; Gouin, T.; Mackay, D.; Matthies, M., Temperature dependence of the characteristic travel distance. *Environ. Sci. Technol.* **2003,** *37*, (4), 766-771. doi:10.1021/es025717w.
24. Wania, F.; Daly, G. L., Estimating the contribution of degradation in air and deposition to the deep sea to the global loss of PCBs. *Atmospheric Environment* **2002,** *36*, (36-37), 5581-5593.
25. Shen, L.; Wania, F., Compilation, evaluation, and selection of physical-chemical property data for organochlorine pesticides. *Journal of Chemical and Engineering Data* **2005,** *50*, (3), 742-768. doi:10.1021/je049693f.
